# Synergistic Effects of Microplastics and Marine Pollutants on the Destabilization of Lipid Bilayers

**DOI:** 10.1021/acs.jpcb.4c03290

**Published:** 2024-09-02

**Authors:** Jean-Baptiste Fleury, Vladimir A. Baulin

**Affiliations:** † Experimental Physics and Center for Biophysics, Universitat des Saarlandes, 66123 Saarbruecken, Germany; ‡ Departament d’Enginyeria Química, Universitat Rovira i Virgili, Av. dels Països Catalans, 26, 43007 Tarragona, Spain

## Abstract

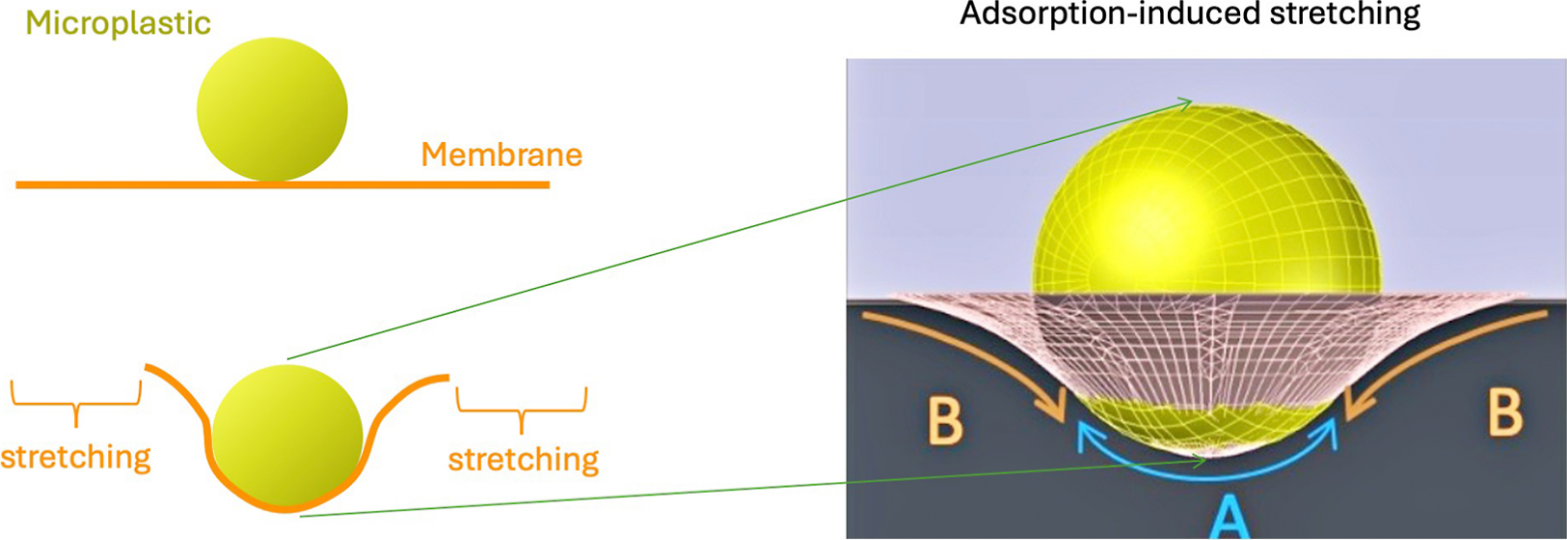

Microplastics have been detected in diverse environments, including soil, snowcapped mountains, and even within human organs and blood. These findings have sparked extensive research into the health implications of microplastics for living organisms. Recent studies have shown that microplastics can adsorb onto lipid membranes and induce mechanical stress. In controlled laboratory conditions, the behavior and effects of microplastics can differ markedly from those in natural environments. In this study, we investigate how exposure of microplastics to pollutants affects their interactions with lipid bilayers. Our findings reveal that pollutants, such as chemical solvents, significantly enhance the mechanical stretching effects of microplastics. This suggests that microplastics can act as vectors for harmful pollutants, facilitating their penetration through lipid membranes and thus strongly affect their biophysical properties. This research underscores the complex interplay between microplastics and environmental contaminants.

## Introduction

The widespread proliferation of plastics has led to an alarming increase in plastic waste, much of which ends up in the oceans. This waste undergoes fragmentation into micro- and nanoplastics,^1^ which pose significant environmental and health risks due to their ability to cross physiological barriers and disrupt biological functions.^2−4^ Plastic became one of the first sources of ocean pollution from human industrial production.^5,6^ Among them, the greatest concern relates to microplastics: tiny plastic particles with a broad range of sizes between ≈0.1 μm and 5 mm.^7,8^ Microplastics may be carried into the atmosphere through evaporation due to their small size and physical characteristics,^9,10^ where they are spread out evenly everywhere when it rains or snows.^11,12^ Marine mammals, particularly whales, ingest colossal amounts of microplastics through their prey.^13^ Blue whales, which feed almost exclusively on krill, ingest an estimated 10 million microplastic pieces per day. Fin whales, which feed on both krill and fish, ingest an estimated 3–10 million microplastic pieces per day. Humpback whales that primarily ingest fish ingest an estimated 200,000 pieces of microplastic per day, while those eating mostly krill ingest at least 1 million pieces. Microplastics have been detected in human blood and organs. Therefore, these pollutants present a significant risk to human health that is not yet fully understood.^14−18^ Microplastic particles are rarely directly responsible for the death of living organisms.^19^ However, they may have an impact on cellular and subcellular levels.^20,21^ For instance, they may trigger oxidative stress, membrane damage, an immunological response, or tissue inflammation that results in cellular toxicity.^22,23^ Such effects are, in general, mediated by biological or chemical pathways.^22,23^ However, the presence of microplastics may also cause significant cell membrane instability through purely physical means.^24^ For example, mechanical stretching applied by the microplastics on the cell membrane can destabilize the membranes.^24−26^

Apart from microplastics, there are a myriad of chemical components that can interact with microplastics in the environment and seawater.^27^ In the following, we briefly present four important pollutant families that can be found in the oceans. These four families of contaminants are endocrine disrupting chemicals (EDCs), heavy metals, persistent organic pollutants (POPs), and commercial sunscreen. Mercury and other heavy metals can reach the ocean, mainly as a result of industrial activity, air deposition, and rock erosion.^28−30^ Complex chemical mixtures in the ocean include many EDCs.^31−33^ A common type of EDC is a pesticide, and one of the most historically used was dichlorodiphenyltrichloroethane (DDT).^34−37^ For this reason, DDT is one of the pesticides most present in the oceans.^34−37^ A third family of common marine pollutants are POPs, which are toxic carbon-based compounds that have contaminated the oceans and marine ecosystems.^38,39^ Examples of POPs are perfluoroalkyles, perfluorosurfactant, aliphatic hydrocarbon, and aromatic hydrocarbon.^38^ Commercial sunscreens are also considered as a category of common chemical pollutants.^40−42^

In the following section, we look into how chemical pollution affects the physical interactions between lipid membranes and microplastics. We concentrate our discussion on the forms of microplastics that are primarily found in the oceans because unique interactions between microplastics and cells depend on their size and chemical composition.^43^ The average microplastic size distribution is estimated at ≈0.1 μm to ≈5 mm.^7,8^ As a result, we only used spherical microplastics with a diameter of ≈1 μm. Since real microplastics can have extremely complicated geometrical shapes, we restrict our investigation to the situation of spherical objects for simplicity.^7,8^ The most widely used microplastics are made of acrylics, polypropylene, polyethylene (PE), polystyrene (PS), and polyamide (PA).^8,44^ In this study, we focus on microplastics made of PE, PS, or PA as a material.

## Materials and Methods

### Molecules and Microplastics

In this paper, the formed bilayers have a DOPC/DOPE lipid composition (60:40 in molar ratio).^45,46^ DOPC is the abbreviation of 1,2-dioleoyl-*sn*-glycero-3-phosphocholine, and DOPE is 1,2-dioleoyl-*sn*-glycero-3-phosphoethanolamine. This lipidic composition is different than the one used in ref (24). This is because the bilayer presents a lower tension for this lipidic composition. This is mandatory, as the presence of pollutants increases the bilayer tension. Thus, we employed a composition with a lower bilayer tension to allow measurement in the presence of microplastics contaminated with pollutants. We also used purchased DOPE-Atto647N is 1,2-dioleoyl-*sn*-glycero-3-phosphoethanolamine labeled with Atto 647N. All the lipids were purchased from Avanti Polar Lipids (USA). As heavy metal we used mercury, as POPs we used hexane (CAS 110-54-3), toluene (CAS 108-88-3), 1-octanol (CAS 111-87-5), perfluoroctanol (CAS 647-42-7), and zonyl (CAS 65545-80-4), as EDC we employed DDT (CAS 50-29-3) and all these products were purchased from Sigma-Aldrich. Sunscreen were purchased from several commercial sunscreen products available in supermarket. Filtrated seawater was purchased from Holoslife. Due to the quantity and pollutants chemical nature, we can ignore plastic dissolution.^47^

Three different types of microplastic beads were used: PS (diameter 0.8 μm), PE (1 μm), and polymethacrylate (PMMA) (1 μm). PS microbeads (Bangs Lab, USA, 0.798 μm, Shamrock Green uniformly dyed; catalog number: DSSG005). PE microbeads (Cospheric, catalog number CPMS-0.96) diameters from 1 to 10 μm (filtered to ≈1 μm by a standard microfluidic filter). PMMA microbeads, as an alternative to PA (Sigma-Aldrich, catalog number 90875) (1 μm). Red fluorescent polystyrene microplastic beads (PS) were purchased from Thermo Fisher (R0100), with a diameter 1 μm. Each of them, were dried from their solution, dispersed in ethanol to remove the surfactant, and extracted via centrifuging (3000 rpm during 10 min). The microplastics were redispersed in pure water. The process of surfactant removal was repeated three times.

### Surface Tension Measurements

Surface tension of various lipid monolayers at the oil–water interface was obtained by the pendant drop method using a commercial measurement device (OCA 20, DataPhysics Instruments GmbH, Filderstadt, Germany). An oil solution containing 5 mg/mL of lipids was produced by introducing a droplet from a steel needle into the surrounding oil phase. The interfacial tension was obtained from fitting of the shapes of the droplets by the Young–Laplace equation.^48^

### Droplet Interface Bilayers Fabrication

Lipids were dissolved in squalene oil at a concentration of 5 mg/mL. The lipids were left for 24 h at 50 °C under magnetic stirring. The OTS-coated glass container, which has a cylinder shape that is 1 cm in height and has a diameter of 7 cm, was filled with the oil–lipid mixture. This device was placed on a hot plate and disposed at the desired temperature. A large area of the cylinder can be observed by reflection using a Leica Z16 Microscope connected to a PCO1600 camera. The optical quality is reduced when using this technique. However, it is enough to distinguish DiB [droplet interface bilayer (DiB)] that have merged from others. For formation and manipulation of an aqueous microdroplet, a micropipette with a desired tip, having a typical diameter in the range 1 mm, was formed using a micropipette puller (Eppendor). Using this method, two water droplets of nearly equal size are produced manually in this container and left at rest for 30 min. They are gently brought into contact via a needle. After a few minutes, a bilayer appears spontaneously at the contact area between the droplets.^24,49,50^ The buffer composition of each droplet was determined before droplet production. Thus, a controlled amount of microplastics could be dissolved into the buffer prior to droplet production.

### Microfluidic Free-Standing Bilayer Fabrication

A 3D microfluidic chip was used to produce a horizontal bilayer. To produce a horizontal bilayer, we produced two molds by using the 3D printing technique and used it to mold a polydimethylsiloxane (PDMS—Sylgard 184—Dow Corning) block. The PDMS block was plasma bound to a glass coverslip after plasma treatment (Diener). This technique is described in more detail in the following refs (24, 46, 51, and 52). Then an oil–lipid mixture was injected into this chip until it filled the chip. Lipids were dissolved in squalene oil at a concentration of 5 mg/mL. The lipids were left for 24 h at 50 °C under magnetic stirring prior to injection into the chip. Then, two buffer phases were injected face-to-face until they met at a desire location. Each water–oil interface was covered by a lipid monolayer and after the two monolayers were brought into contact to produce a bilayer.^24,46,51,52^

### Small Unilamellar Vesicles

We dispersed 2.6 mM total phospholipids in a glass test tube, using the fixed molar ratio of phospholipids (e.g., 78 mol % DOPC, 20 mol % DOPS, 2% Atto647N-DOPE) in 1 mL of chloroform (Sigma). The mixture was then dried with nitrogen and dispersed in 2 mL of phosphate-buffered saline (PBS) (using several falcons). Then, using a Vibracel titanium-tip sonicator (Bandeli, Sonopuls, Germany) with a maximum power of 600 W and frequency of 20 kHz, ultrasonic radiation was applied to this mixture. Each sample underwent repetitive 3 Hz cycles that consisted of 1 s pulses at a power of 150 W to control the thermal effects. Finally, these samples were placed in the fridge for 1 day.

### Imaging and Particle Tracking

Fluorescent movies of the beads on the lipid bilayer were recorded by using an Axio Z7 Observer microscope (Zeiss). The microscope was equipped with a Colibri 7 LED illumination system, which provided stable and uniform lighting necessary for high-quality fluorescence imaging. The setup included appropriate filters and objectives to ensure optimal excitation and emission wavelengths for the fluorescent beads.

For tracking beads on the lipid bilayer surface, we utilized a 3D microfluidic setup, as described in previous studies.^25,46^ This setup allows for the precise manipulation and observation of microscale particles within a controlled environment. The microfluidic device was fabricated by using standard soft lithography techniques, ensuring accurate channel dimensions and uniform flow conditions.

### Image Analysis

The analysis of particle tracking data was performed using Python programming language and the TrackPy library.^53^ TrackPy is an open-source software package designed for tracking the positions of particles in video data. The analysis pipeline included the following steps: (i) *Particle detection*: the positions of the beads were detected in each frame using TrackPy’s built-in functions, which apply image processing techniques to identify and localize particles. (ii) *Trajectory linking*: the detected positions were linked frame-by-frame to construct the trajectories of individual beads. This step accounts for potential particle movements between frames and ensures continuous tracking over time. (iii) *Data analysis*: the trajectories were analyzed to extract diffusion coefficients, mean square displacement (MSD), and velocity distributions.

## Results and Discussion

**Figure 1:**
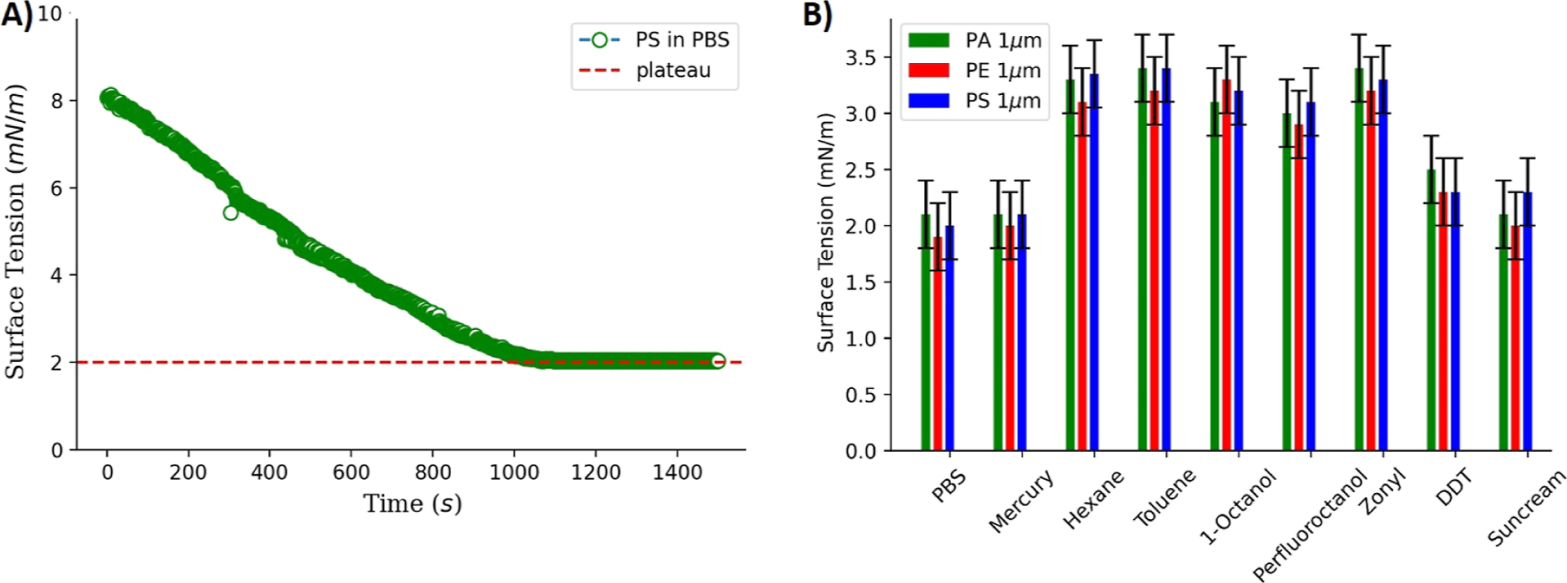
(A) One example of surface tension measurement from the water pendant method. The dashed line indicates the surface tension plateau, which is the measured value. The pendant droplet consists of a PBS buffer and its shape is analyzed as a function of time (s). The buffer droplet was produced in an oily phase (squalene), which contains phospholipids. For this measurement, PE beads were dispersed in the buffer droplet at a concentration of 50 μg/mL. (B) Surface tension measurements using the water pendant droplet method (see Materials and Methods section) and a fixed microplastics concentration of approximately *c* ≈ 50 μg/mL are plotted after incubation in seawater with different marine pollutants.

The microplastics were initially washed with ethanol to remove any surfactant residue that may have been used to stabilize the microplastics in the solution (refer to the Materials and Methods section). Subsequently, 0.1 mg/mL of microplastics were incubated in 2 mL of seawater containing a single type of pollutant in a glass falcon, and placed on a shelf for a month without light. Following this incubation period, the microplastics were extracted via centrifugation. They were then thoroughly washed with pure water and ultimately dispersed in a PBS solution to achieve a final concentration of 0.5 mg/mL. This model process of microplastics treatment mimics the microplastics’ destiny in the environment: they are floating in the ocean and fragmenting until they evaporate into the atmosphere and are inhaled or absorbed by living beings and make contact with cell membranes. The following pollutants were utilized during incubation: 0.639 ppm/L for mercury, 0.526 mg/L for toluene, 25 μg/L for DDT, 1.3 g/mL for perfluoroctanol and 1-octanol, 0.014% of volume for hexane, 1.1 g/mL of zonyl, and 1 mg/mL of a mixture from numerous commercial sunscreens. Except for sunscreen and perfluoroctanol, these values correspond to their maximum solubility in pure water, although we can assume their solubility is probably slightly less in seawater. In summary, we studied one heavy metal (mercury), four POPs (hexane, toluene, 1-octanol, perfluoroctanol, and zonyl), one EDC (DDT), and several sunscreens. Using the water pendant droplet method, these microplastics were used to measure the surface tension γ of the phospholipid monolayer at the water/squalene oil interface (see the Materials and Methods section).^48^ We kept the concentration of microplastics *c* ≈ 50 μg/mL constant throughout these measurements (see Figure 1). The values in PBS correspond to the control surface tension γ values (γ ≈ 2 mN/m). At this condition, the microplastics were incubated in filtrated seawater from the Mediterranean Sea without any pollutants before being washed out and dispersed in PBS. These values do not appear to be impacted by the presence of mercury. DDT and sunscreen induced a slight increase in the measured surface tension values (γ ≈ 2–2.3 mN/m). However, a more significant increase is measured for zonyl, hexane, toluene, 1-octanol, and perfluoroctanol (γ ≈ 3 mN/m). These measurements indicate that POPs can modify the surface properties of microplastics, which can then alter the adsorption and interaction of microplastics with lipid bilayers and other biological surfaces. Thus, the specific chemical interactions between the terminal groups on the model microspheres (PA, PE, PS) and the POPs likely play a role in the different surface tension effects observed in Figure 1B. More polar terminal groups of PA microspheres may interact more strongly with certain POPs compared to the less polar PE or PS microspheres. Additionally, the size and crystallinity of the microplastics can also affect their interactions with POPs.

**Figure 2:**
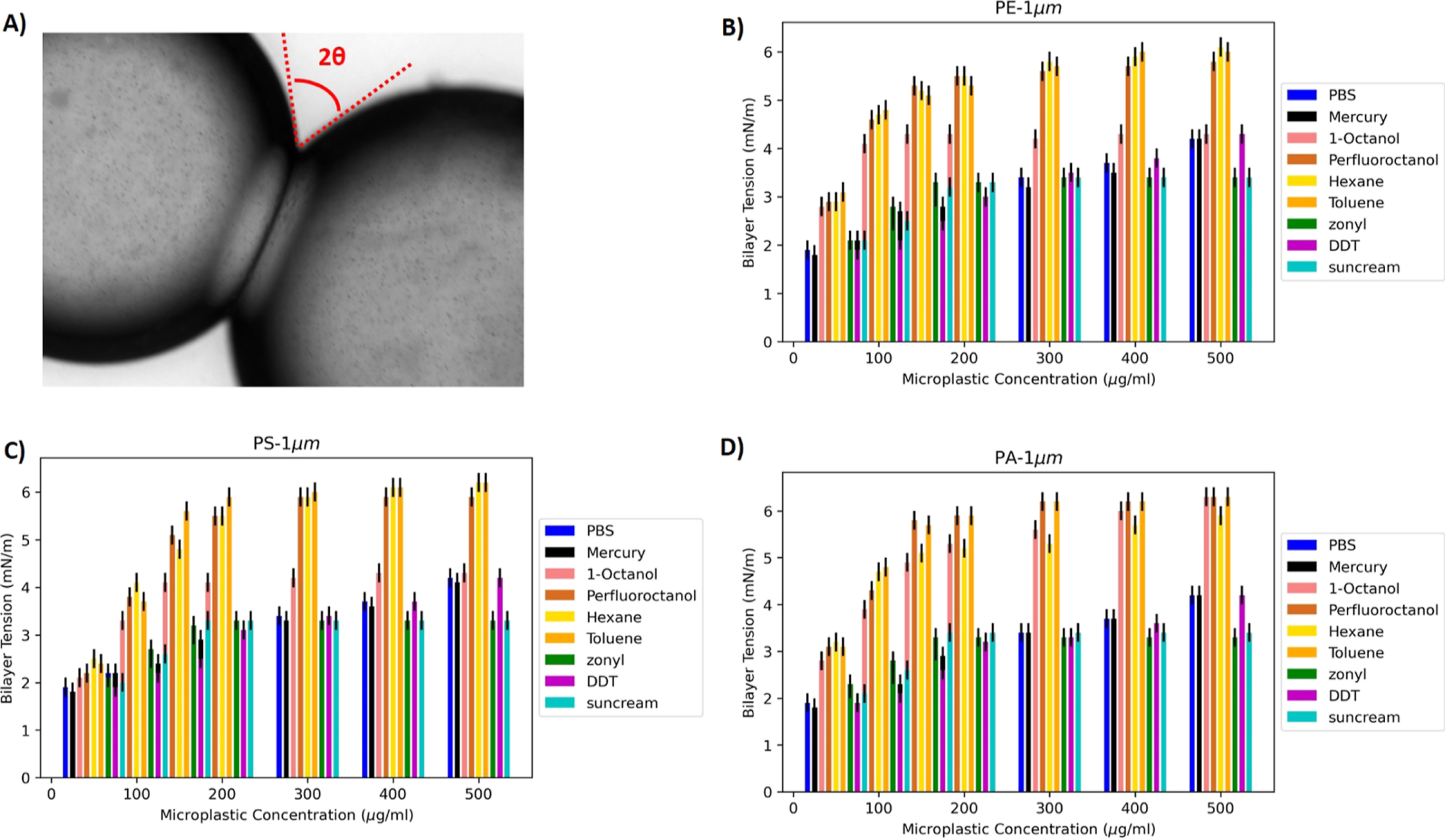
(A) A microscopic picture of a bilayer formed by the DiB method. The two buffer droplets are made of PBS and contain 100 μm/mL PE beads. (B) Present bilayer tension measurements obtained from the Young–Dupré equation, as a function of different 1 μm PE microplastics concentration after incubation in seawater with different marine pollutants. The reported bilayer tension values are obtained from an average of ≈20–30 measurements. (B,C) Are the same measurements as reported for (A), except that they correspond to PS and PA microplastics, respectively.

Using these surface tension data, we can now calculate the bilayer tension in the presence of microplastics incubated with various marine pollutants. For this purpose, we employ the DiB technique to produce free-standing lipid bilayers.^50^ In this technique, two water droplets of comparable size are formed in an oily phase that contains phospholipid (see the Materials and Methods section). Each water–oil interface is decorated by a lipid monolayer, and a bilayer is formed when these two droplets come into contact. Under a microscope, a visual optical confirmation of the bilayer formation may be seen, and the Young–Dupré law can be utilized to determine the associated bilayer tension Γ^48^
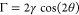
1where 2θ is the contact angle measured by the DiB method (see Figure 2 and ref (50)). One benefit of the DiB approach is that it enables the high-throughput creation of bilayers and avoids issues with sedimentation or buoyancy (the bilayer is vertical).^24^Tables 1 and 3 list the measured tensions for various types of microplastics and concentrations (see Table 2).

**Table 1:** Bilayer Tension of PS Microplastics Measured in the Presence of Marine Pollutants as a Function of Microplastic Concentration *c*^a^

chemicals	PS—bilayer tension Γ (mN/m)					
	0 μg/mL	50 μg/mL	100 μg/mL	150 μg/mL	300 μg/mL	500 μg/mL
PBS	1.7 ± 0.2	1.9 ± 0.2	2.2 ± 0.2	2.5 ± 0.3	3.4 ± 0.3	4.2 ± 0.4
mercury	1.7 ± 0.2	1.8 ± 0.2	2.2 ± 0.2	2.4 ± 0.3	3.3 ± 0.3	4.1 ± 0.4
hexane	1.7 ± 0.2	2.5 ± 0.5	4.1 ± 0.5	4.8 ± 0.5	5.9 ± 0.5	6.2 ± 0.5
toluene	1.7 ± 0.2	2.4 ± 0.5	3.7 ± 0.5	5.6 ± 0.5	5.9 ± 0.5	6.2 ± 0.5
1-octanol	1.7 ± 0.2	2.18 ± 0.5	3.3 ± 0.5	4.3 ± 0.5	4.3 ± 0.5	4.3 ± 0.5
perfluoroctanol	1.7 ± 0.2	2.2 ± 0.4	3.8 ± 0.4	5.1 ± 0.5	5.9 ± 0.5	5.9 ± 0.5
zonyl	1.7 ± 0.2	2.1 ± 0.3	2.7 ± 0.3	3.2 ± 0.4	3.3 ± 0.4	3.3 ± 0.5
DDT	1.7 ± 0.2	1.9 ± 0.2	2.2 ± 0.2	2.5 ± 0.3	3.4 ± 0.3	4.2 ± 0.4
sunscreen	1.7 ± 0.2	2 ± 0.3	2.6 ± 0.3	3.3 ± 0.4	3.3 ± 0.4	3.3 ± 0.5

a The reported values are obtained by averaging of approximately 20–30 measurements. The microplastics were incubated in filtered sea water without any pollutants before being washed out and dispersed in the PBS.

**Table 2:** Table Summarizes the Bilayer Tension for PE Microplastics Measured in the Presence of Marine Pollutants and as a Function of Microplastic Concentration *c*^a^

chemicals	PE—bilayer tension Γ (mN/m)					
	0 μg/mL	50 μg/mL	100 μg/mL	150 μg/mL	300 μg/mL	500 μg/mL
PBS	1.7 ± 0.2	1.9 ± 0.2	2.1 ± 0.2	2.5 ± 0.3	3.4 ± 0.3	4.2 ± 0.4
mercury	1.7 ± 0.2	1.8 ± 0.2	2.1 ± 0.2	2.7 ± 0.3	3.2 ± 0.3	4.2 ± 0.4
hexane	1.7 ± 0.2	2.9 ± 0.5	4.7 ± 0.5	5.2 ± 0.5	5.8 ± 0.5	6.1 ± 0.5
toluene	1.7 ± 0.2	3.1 ± 0.5	4.8 ± 0.5	5.1 ± 0.5	5.7 ± 0.5	6 ± 0.5
1-octanol	1.7 ± 0.2	2.8 ± 0.5	4.1 ± 0.5	4.3 ± 0.5	4.2 ± 0.5	4.3 ± 0.5
perfluoroctanol	1.7 ± 0.2	2.9 ± 0.4	4.6 ± 0.4	5.3 ± 0.5	5.6 ± 0.5	5.8 ± 0.5
zonyl	1.7 ± 0.2	2.1 ± 0.3	2.8 ± 0.3	3.3 ± 0.4	3.4 ± 0.4	3.4 ± 0.5
DDT	1.7 ± 0.2	1.9 ± 0.2	2.1 ± 0.2	2.5 ± 0.3	3.5 ± 0.3	4.3 ± 0.4
sunscreen	1.7 ± 0.2	2.1 ± 0.3	2.5 ± 0.3	3.2 ± 0.4	3.4 ± 0.4	3.4 ± 0.5

a The reported bilayer tension values are obtained from an average of approximately 20–30 measurements. For the PBS, it means that the microplastics were incubated in filtrated sea water without any pollutants before being washed out and dispersed in the PBS.

**Table 3:** Table Summarizes the Bilayer Tension for PA Microplastics Measured in the Presence of Marine Pollutants and as a Function of Microplastic Concentration *c*^a^

chemicals	PA—bilayer tension Γ (mN/m)					
	0 μg/mL	50 μg/mL	100 μg/mL	150 μg/mL	300 μg/mL	500 μg/mL
PBS	1.7 ± 0.2	1.9 ± 0.2	2.2 ± 0.2	2.5 ± 0.3	3.4 ± 0.3	4.2 ± 0.4
mercury	1.7 ± 0.2	1.8 ± 0.2	1.9 ± 0.2	2.3 ± 0.3	3.4 ± 0.3	4.2 ± 0.4
hexane	1.7 ± 0.2	3.2 ± 0.5	4.7 ± 0.5	5.1 ± 0.5	5.3 ± 0.5	5.9 ± 0.5
toluene	1.7 ± 0.2	3.1 ± 0.5	4.8 ± 0.5	5.7 ± 0.5	6.2 ± 0.5	6.3 ± 0.5
1-octanol	1.7 ± 0.2	2.8 ± 0.5	3.9 ± 0.5	4.9 ± 0.5	5.6 ± 0.5	6.3 ± 0.5
perfluoroctanol	1.7 ± 0.2	3.1 ± 0.4	4.3 ± 0.4	5.8 ± 0.5	6.2 ± 0.5	6.3 ± 0.5
zonyl	1.7 ± 0.2	2.3 ± 0.3	2.8 ± 0.3	3.3 ± 0.4	3.2 ± 0.4	3.3 ± 0.5
DDT	1.7 ± 0.2	2.6 ± 0.2	3.2 ± 0.2	3.3 ± 0.3	3.5 ± 0.3	4.2 ± 0.4
sunscreen	1.7 ± 0.2	2.1 ± 0.3	2.6 ± 0.3	3.4 ± 0.4	3.4 ± 0.4	3.4 ± 0.5

a The reported bilayer tension values are obtained from an average of approximately 20–30 measurements. For the PBS, it means that the microplastics were incubated in filtrated sea water without any pollutants before being washed out and dispersed in the PBS.

The bilayer tension increased with the concentration of the microplastics. PS microplastics incubated in pure seawater and redispersed in PBS show an increase in bilayer tension Γ from ≈2 mN/m for *c* ≈ 50 μg/mL to Γ ≈ 4 mN/m for *c* ≈ 500 μg/mL. This behavior is similar for PS, PA, and PE microplastics that were incubated with mercury and DDT. PS microplastics incubated with sunscreen or zonyl are showing a slightly increasing bilayer tension from Γ ≈ 2 mN/m for *c* ≈ 50 μ g/mL to Γ ≈ 3–4 mN/m for *c* ≈ 500 μg/mL (see Figure 2). This slight increase is also notable in the case of PE and PA microplastics. The most striking bilayer tension increase was measured for all the microplastics incubated with POPs (hexane, toluene, 1-octanol, and perfluoroctanol), where the bilayer tensions are increasing from Γ ≈ 2 mN/m for *c* ≈ 50 μg/mL to Γ ≈ 4–6 mN/m for *c* ≈ 500 μg/m. Similar behavior for PA and PS microplastics with the POPs pollutants again shows the most striking increase in bilayer tension. Where PA and PE microplastics present values from Γ ≈ 2 mN/m for *c* ≈ 50 μg/mL to Γ ≈ 4–6 mN/m for *c* ≈ 500 μg/mL (see Figure 2).

In order to investigate the nature of physical interaction between microplastics and a lipid bilayer, we produced a horizontal bilayer and dispersed fluorescent PS microplastics around it (Figure 4A). It appears that these microplastics diffuse continuously on the bilayer surface and do not become immobile after touching it. We track and analyze these microplastics’ motions in order to determine their MSD ⟨*r*^2^⟩ and the corresponding diffusion constant . The measured diffusion constant *D* ≈ 0.6 μm^2^ s^–1^ is close to the bulk diffusion value for a 1-μm microplastic (see Figure 4B,C).^24,55^ This characteristic motility and diffusion stay unchanged for all of the considered microplastics in this article.

Figure 4C shows the effect of microplastics incubated with hexane on the bilayer tension for different concentrations of microplastics. The curve gradually increases with the same law as in the absence of pollutants. This result suggests that the diffusion properties of the incubated microplastics are not quantitatively changed by the presence of hexane.^24^ However, incubation with hexane and other hydrophobic molecules increases the bilayer tension. The adsorption of hydrophobic molecules of perfluorooctanol, hexane, and toluene on the microplastic surface changes the interfacial interactions between the microplastics and the lipid bilayer as they prefer to insert into the hydrophobic core of the bilayer.^56^ This can lead to a mismatch between the microplastic surface properties and the lipid bilayer, leading to an expansion of the bilayer and an increase in its thickness and causing the bilayer to become stretched or deformed and, thus, resulting in an increase in the overall bilayer tension. This suggests stronger destabilization of the lipid bilayer in the presence of a pollutant and stronger mechanical deformation of the lipid bilayer. In contrast, DDT has a large, rigid structure due to its aromatic rings and multiple chlorine atoms. This structure makes it less likely to insert smoothly into the lipid bilayer and less likely to increase the surface tension. Its size and shape can limit its mobility within the bilayer, reducing its ability to disrupt lipid packing and increase the bilayer tension. Mercury due to its metallic nature does not integrate well into the lipid bilayer, and it does not interact with lipid molecules in a way that would disrupt their packing and increase tension.

**Figure 3:**
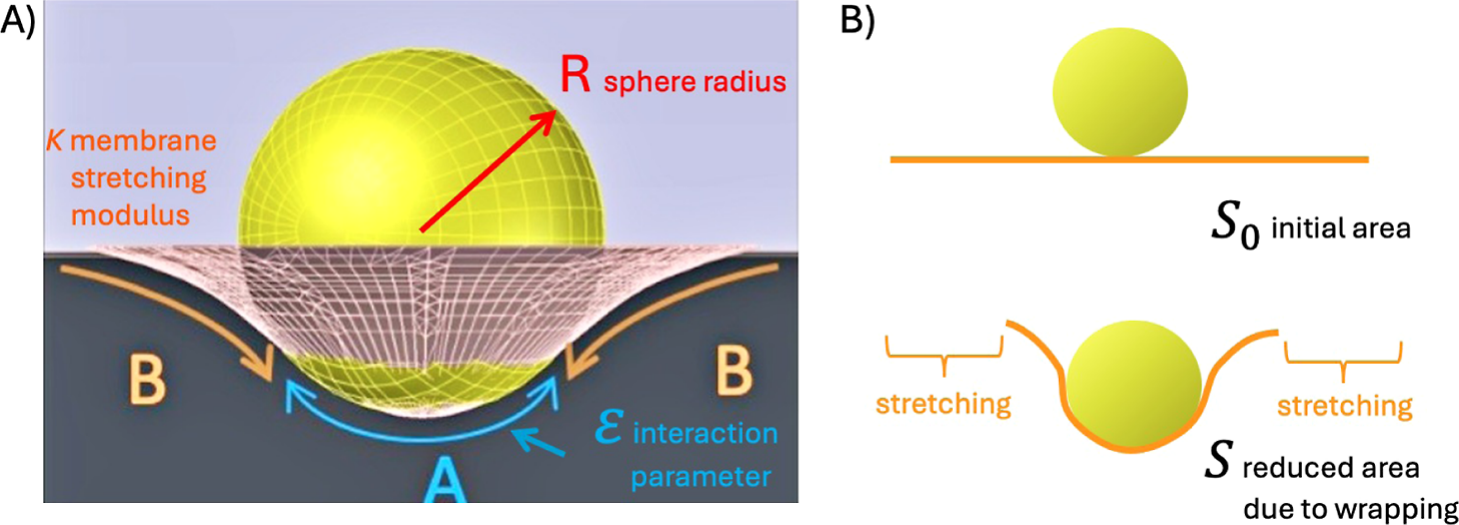
(A) Interaction of an individual microplastic sphere of radius *R* with a lipid membrane. The adsorption region A is characterized by the interaction parameter ϵ, while the free region B is characterized by the membrane stretching modulus *k*. (B) The mechanism of stretching due to crampling of the surface by a sphere.

To understand the effects of pollutants on the stretching of the lipid bilayer, we used the same elastic layer model of a lipid bilayer interacting with bare spherical microplastics described in ref (24). This model describes the mechanical stretching of the lipid bilayer due to the adsorption of microplastics and the resulting local deformation of the lipid bilayer around microplastics. A similar model was used to stretch cell membranes^57^ with nanopillars and to mechanically deform bacteria cell membranes with gold nanoparticles.^26^ Within this model, the area available per microplastic particle is split into two parts: the free-standing or “suspended” part A and the “adsorbed” part B; see Figure 3. The balance of the stretching/compression of the layers A and B and the attraction to the microplastics in the contact region A determines the equilibrium position of the layer. In the case of the adsorption of the particles onto the membrane, the free-standing region A is stretched to increase the contact of the membrane with the particle in region B (Figure 3A). Thus, the free energy of the membrane is the sum of two terms: the energy gain due to adsorption in the region B and the membrane stretching in the region A^57^
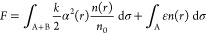
2where ε is the dimensionless interaction parameter between the surface of the particle and the membrane, *k* is the compressibility constant, α = (*S* – *S*_0_)/*S*_0_ is the dimensionless parameter describing local stretching, *S* is the actual area and *S*_0_ is the equilibrium unperturbed area before the contact with the particle (Figure 3B). *n*(*r*) = *n*_0_/(1 + α(*r*)) and *n*_0_ are the local density of adsorption points at position *r* and in the unperturbed membrane, correspondingly. This free energy is then minimized with the constraint of the conservation of the total area.

As a result, the set of nonlinear equations for the areas in regions A and B provides the stretching in both regions as a function of adsorption strength. Within this model, the mechanical stretching is controlled by the ratio
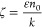
3where ε*n*_0_ is the attractive interaction energy per area. The estimates from experimental measurements of the adhesion energies of PS microparticles are of the order 1 mJ/m^2^,^58,59^ while the compressibility constant of a lipid bilayer is *k* ∼ 100–300 mN/m. This gives the range of the control interaction parameter ζ = −0.003 to −0.01 for bare plastics.

**Figure 4:**
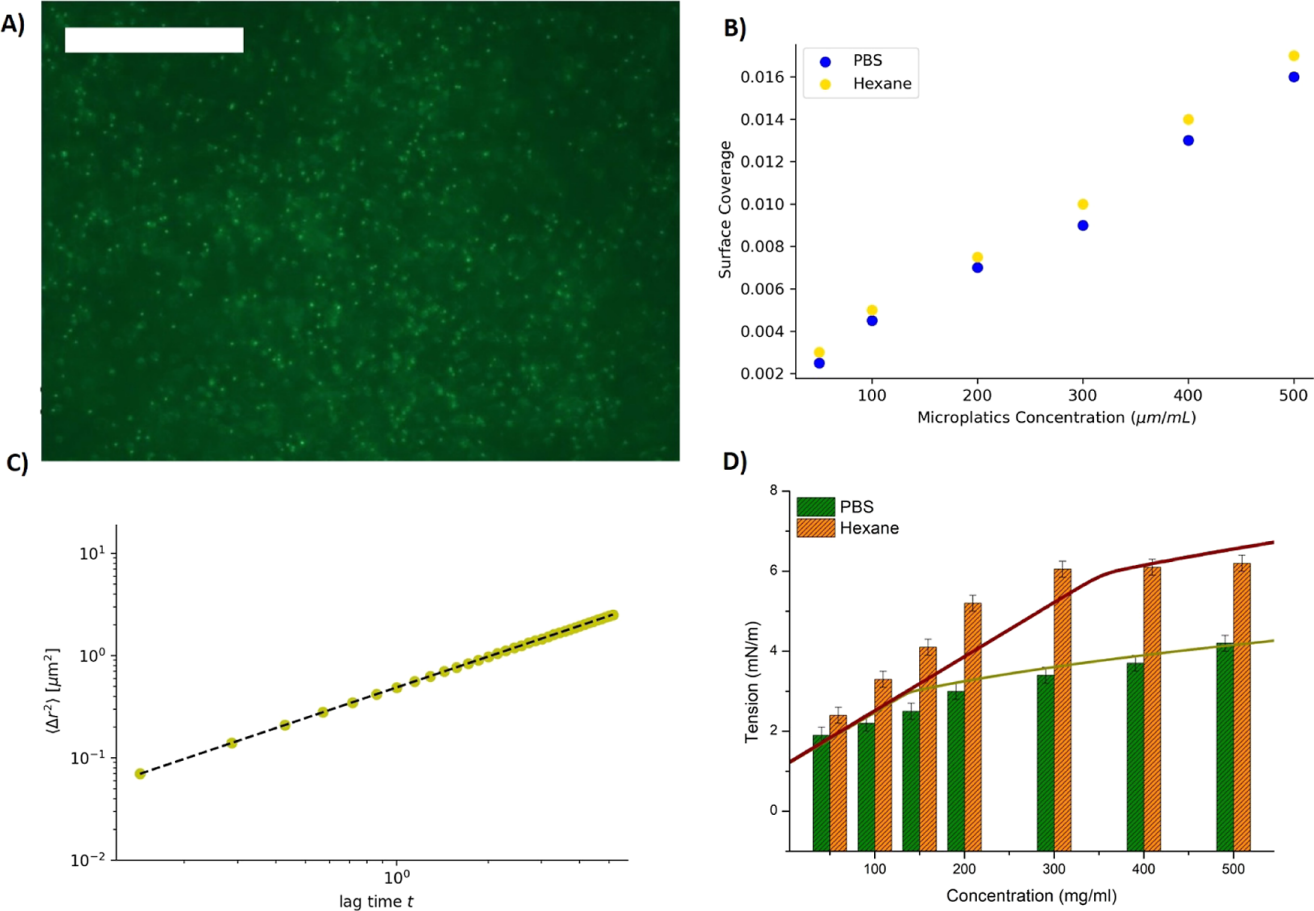
(A) A micrograph of red fluorescent 1 μm PS microplastics adsorbed on a free-standing lipid bilayer (bar is 100 μm). (B) The adsorption isotherm of 1 μ m PS (yellow circles) microplastics, incubated in seawater with 0.014% volume of hexane, adsorbed on a free-standing lipid bilayer. Blue circles are plots for 1 μm PS microplastics incubated in seawater plus hexane, which are adsorbed on a free-standing lipid bilayer. (C) The extracted MSD as a function of time on a log–log scale, for a 1 μm PS incubated in hexane. The measured average microplastic diffusion coefficient *D* ≈ 0.6 μm^2^·s^–1^. (D) Bilayer tensions measured similar to Figure 2C, except that PS microplastics were incubated in seawater in the presence of hexane. The experimental data are represented as bar plot, while the continuous line represents the theoretical plot. It was obtained using the surface coverage data plotted in (B).

The experimental tension of bare microplastics in PBS (Figure 4D) can be compared directly with the predicted value from the model, assuming the value of the control parameter ζ = −0.01. The theoretical curve with only one parameter fits well with the experimental data (Figure 4D). The bilayer tension of PS microplastic incubated with hexane can be approximated with the theoretical curve with no additional parameters, if we assume the attraction to the bilayer is 10× stronger, ζ = −0.1. Thus, if qualitatively the physical interaction seems not to have changed, quantitatively we observed a notable increase (10 times for hexane) in the attraction to the bilayer with respect to bare microplastics. The most significant tension increases are measured for microplastics incubated with POPs. As the polluted seawater is washed out and replaced with PBS, we can assume that the quantity of pollutants in the buffer is negligible. Thus, we believe that some pollutant molecules adsorb onto the surface of the microplastics and alter the surface properties, resulting in a significant change in the adsorption strength of the bilayer. These findings go in line with recent studies indicating that POPs may alter the surface properties of microplastics, which may change their adherence to a bilayer.^47,60−63^

**Figure 5:**
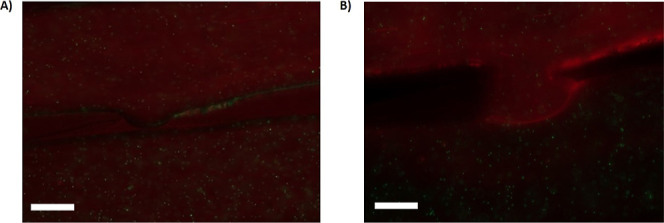
(A) Microfluidic channels to from a vertical free-standing lipid bilayer.^54^ The bilayer was exposed to SUVs (*c* ≈ 1 mM), visible as red dots. The green dots correspond to PS microplastics (1 μ m). (A) The bilayer is in contact with bare PS microplastics, and (B) the bilayer is in contact with PS microplastics contaminated with hexane. The bilayer does not present a fluorescent signal in (A), while the bilayer is fluorescent in (B), which means that the SUVs can fuse in the presence of PS microparticles contaminated with hexane. Bar lengths in panels (A,B) correspond to 200 μm.

In addition, POPs are used as solvents to dissolve phospholipids.^56,64−66^ Due to its amphiphilic nature, these chemicals can even infiltrate the bilayer core and alter its potent biophysical characteristics.^56,64−66^ To examine the ability of microplastics to serve as a vehicle for transport into the lipid bilayers of contaminants such as POPs, a free-standing vertical bilayer was produced in a microfluidic chip.^54^ Hexane-incubated microplastics (*c* ≈ 500 μg/mL) were dispersed near a bilayer for two hours before washing them away from the bilayer. Then we inject small unilamellar vesicles (SUVs) (see the Materials and Methods section, Figure 5) around the bilayer.^67,68^ While there are no fluorescent molecules in the lipid bilayer, these SUVs do contain some fluorescent lipids (DOPE-Atto647N, 2% in molar ratio). We performed tests to ensure that these SUVs are stable and do not fuse or hemifuse with the lipid bilayer in the absence of microplastics. However, following exposure to microplastics incubated in hexane, we see that after scattering SUVs close to the bilayer, the lipid bilayer became fluorescing after 10–20 min. This observation indicates the presence of SUV fusion or hemifusion with the bilayer. This can occur in the presence of fusogenic molecules like hexane, so we assume that microplastics have delivered hexane into the bilayer core.^69^ This demonstrates that microplastics can serve as vectors into a lipid bilayer for chemical molecules.

Finally, we also report a surprising minimal or nonexistent mechanical impact for the other categories of contaminants. For example, we do not observe an increase in tension for mercury and DDT. It may be explained by the low quantities of mercury and the large aromatic rings that have difficulties in inserting into the bilayer. Zonyl is a perfluorated surfactant, so it can have an antagonistic interaction with the oil–water interface. As a surfactant, it may decrease the surface tension of a pure oil–water interface but may also increase bilayer tension once inside a lipid bilayer, which may explain the weak measured tension with zonyl-contaminated microplastics. Notably, our study investigates only physical interactions between microplastics and lipid bilayers; it does not take into account biological or other toxicological pathways associated with marine pollutants.

## Conclusions

In this paper, we investigate the physical interaction between microplastics and a lipid bilayer in the presence of typical marine pollutants. The seawater with added typical marine pollutants is incubated with typical microplastics found in the environment to simulate the contamination of microplastics in seawater closer to real environmental conditions. We let them for one month and dispersed the contaminated microplastics in the PBS. This step models the microplastics’ evaporation from the ocean and their ingestion by a living organism. Using a microfluidic setup, we produced a free-standing lipid bilayer and measured the microplastics’ effect on the bilayer tension. We found that microplastics contaminated by POPs increase the bilayer tension. Using a theoretical model, we could estimate the effective increase in the microplastic adhesion properties in the presence of chemical pollutants. We hypothesize that this higher tension may be the result of a change in the surface characteristics of microplastics subsequent to POP incubation. Moreover, using a custom-made microfluidic fusion assay, we demonstrated that chemical molecules can be vectored by microplastics into the core of lipid bilayers.

While cell membranes and artificial lipid bilayers may differ in their exact lipid composition and membrane components, the fundamental mechanical properties of these membranes are quite similar. This allows the insights gained from studying artificial membranes to be reasonably extrapolated to living cells.

This mechanism of membrane destabilization and potential rupture due to increased tension provides an explanation for how microplastic pollution synergistically combined with the adsorption of hydrophobic compounds can contribute to the toxicity of microplastics to living cells. Our study emphasizes the potential consequences of environmental contamination by chemicals, such as oil spills or industrial accidents.
